# Causal role of immune cells in schizophrenia: Mendelian randomization (MR) study

**DOI:** 10.1186/s12888-023-05081-4

**Published:** 2023-08-15

**Authors:** Chengdong Wang, Dongdong Zhu, Dongjun Zhang, Xiaowei Zuo, Lei Yao, Teng Liu, Xiaodan Ge, Chenlu He, Yuan Zhou, Ziyuan Shen

**Affiliations:** 1 Department of Psychiatry, The Affiliated Xuzhou Oriental Hospital of Xuzhou Medical University, Xuzhou, Jiangsu 221004 China; 2 School of Psychology, Xinxiang Medical University, Xinxiang, Henan 453003 China; 3 Department of Psychiatry, The Second Affiliated Hospital of Xinxiang Medical University, Xinxiang, Henan 453005 China; 4 Department of Epidemiology and Biostatistics, School of Public Health, Xuzhou Medical University, Xuzhou, Jiangsu 221004 China; 5 Medical Technology School of Xuzhou Medical University, Xuzhou, Jiangsu 221004 China; 6 Department of Epidemiology and Biostatistics, School of Public Health, Anhui Medical University, Hefei, Anhui 230032 China

**Keywords:** Schizophrenia, Immunity, Causal inference, Brain, MR analysis, Sensitivity

## Abstract

**Background:**

Complex immune-brain interactions that affect neural development, survival and function might have causal and therapeutic implications for psychiatric illnesses. However, previous studies examining the association between immune inflammation and schizophrenia (SCZ) have yielded inconsistent findings.

**Methods:**

Comprehensive two-sample Mendelian randomization (MR) analysis was performed to determine the causal association between immune cell signatures and SCZ in this study. Based on publicly available genetic data, we explored causal associations between 731 immune cell signatures and SCZ risk. A total of four types of immune signatures (median fluorescence intensities (MFI), relative cell (RC), absolute cell (AC), and morphological parameters (MP)) were included. Comprehensive sensitivity analyses were used to verify the robustness, heterogeneity, and horizontal pleiotropy of the results.

**Results:**

After FDR correction, SCZ had no statistically significant effect on immunophenotypes. It was worth mentioning some phenotypes with unadjusted low *P*-values, including FSC-A on NKT (*β* = 0.119, 95% *CI* = 0.044 ~ 0.194, *P* = 0.002), DN (CD4-CD8-) NKT %T cell (*β* = 0.131, 95% *CI* = 0.054 ~ 0.208, *P* = 9.03 × 10^− 4^), and SSC-A on lymphocytes (*β* = 0.136, 95% *CI* = 0.059 ~ 0.213, *P* = 5.43 × 10^− 4^). The causal effect of SCZ IgD on transitional was estimated to 0.127 (95% *CI* = 0.051 ~ 0.203, *P* = 1.09 × 10^− 3^). SCZ also had a causal effect on IgD+ %B cell (*β* = 0.130, 95% *CI* = 0.054 ~ 0.207, *P* = 8.69 × 10^− 4^), and DP (CD4^+^CD8^+^) %T cell (*β* = 0.131, 95% *CI* = 0.054 ~ 0.207, *P* = 8.05 × 10^− 4^). Furthermore, four immunophenotypes were identified to be significantly associated with SCZ risk: naive CD4^+^ %T cell (*OR* = 0.986, 95% *CI* = 0.979 ~ 0.992, *P* = 1.37 × 10^− 5^), HLA DR on CD14^−^ CD16^−^ (*OR* = 0.738 (95% *CI* = 0.642 ~ 0.849, *P* = 2.00 × 10^− 5^), CD33^dim^ HLA DR^+^ CD11b^−^ AC (*OR* = 0.631, 95% *CI* = 0.529 ~ 0.753, *P* = 3.40 × 10^− 7^) and activated & resting Treg % CD4 Treg (*OR* = 0.937, 95% *CI* = 0.906 ~ 0.970, *P* = 1.96 × 10^− 4^).

**Conclusions:**

Our study has demonstrated the close connection between immune cells and SCZ by genetic means, thus providing guidance for future clinical research.

**Supplementary Information:**

The online version contains supplementary material available at 10.1186/s12888-023-05081-4.

## Introduction

Schizophrenia (SCZ) is a serious psychiatric disorder that often presents with hallucinations, delusions, and extremely disorganized thinking and behavior, which can affect daily physical functioning and may eventually lead to disability [1, 2]. Global Burden of Disease (GBD) research has demonstrated that there are approximately 20.9 million patients with schizophrenia worldwide [3]. At present, antipsychotic medications are the main method of SCZ treatment [4]. SCZ patients often need lifelong treatment, so early diagnosis and treatment may help to control symptoms before serious complications and improve the prognosis [5, 6].

Epidemiologic studies have demonstrated that early-life infection is linked with autoimmune disease and subsequent mental disorders in adults [7–10]. Complex immune-brain interactions that affect neural development, survival, and function might have causal and therapeutic implications for disorders including psychiatric illness [1, 11–13]. Cytokines play an important role in infection and inflammation and are crucial mediators of the crosstalk between the brain and the immune system. SCZ leads to a decrease in T helper type 1 (Th1) and an increase in T helper type 2(Th2) cytokine secretion, which are associated with an imbalance in inflammatory cytokines [14]. Proinflammatory cytokines, which are produced at the site of infection by activated accessory immune cells, leading to endocrine, autonomic and behavior changes, include interleukin-1α and β (IL-1α and IL-1β), tumor necrosis factor-α (TNF-α) and interleukin-6 (IL-6) [12]. A systematic review revealed increased soluble interleukin 2 receptor (SIL-2R), and IL-6 and decreased interleukin-2 (IL-2) in SCZ patients, with no significant differences in other cytokines, compared with the general population [14]. However, findings concerning the association between immune inflammation and SCZ have been inconsistent thus far, possibly due to limited sample sizes, flaws in study design, and confounding factors beyond the scope of the existing studies [15–18].

Mendelian randomization (MR) is an analytical method mainly used in epidemiological etiology inference that is based on Mendelian independent distribution law. It is of great importance that the causal sequence of MR is reasonable [19, 20]. Previous observational studies have found many associations between immune cell traits and SCZ, proving the hypothesis of a correlation between them [21, 22]. In this study, a comprehensive two-sample MR analysis was performed to determine the causal association between immune cell signatures and SCZ.

## Materials and methods

### Study design

We assessed the causal relationship between 731 immune cell signatures (7 groups) and schizophrenia based on a two-sample MR analysis. MR uses genetic variation to represent risk factors, and therefore, valid instrumental variables (IVs) in causal inference must satisfy three key assumptions: (1) genetic variation is directly associated with exposure; (2) genetic variation is not associated with possible confounders between exposure and outcome; and (3) genetic variation does not affect outcome through pathways other than exposure. The studies included in our analysis were approved by the relevant institutional review boards, and participants provided informed consent.

### Genome-wide association study (GWAS) data sources for SCZ

GWAS summary statistics for SCZ were obtained from the Psychiatric Genomics Consortium (PGC) [23]. The study performed a GWAS on 150,064 European individuals (*N*_case_ = 36,989, *N*_control_ = 113,075), with approximately 9.5 million variants analyzed after quality control and imputation. This GWAS identified 128 independent single nucleotide polymorphisms (SNPs) (83 not previously reported), including at least 108 independent genomic loci under the level of genome-wide significance (*P* < 5 × 10^− 8^).

### Immunity-wide GWAS data sources

GWAS summary statistics for each immune trait are publicly available from the GWAS Catalog (accession numbers from GCST0001391 to GCST0002121) [22]. A total of 731 immunophenotypes including absolute cell (AC) counts (n = 118), median fluorescence intensities (MFI) reflecting surface antigen levels (n = 389), morphological parameters [MP] (n = 32) and relative cell (RC) counts (n = 192) were included. Specifically, the MFI, AC and RC features contain B cells, CDCs, mature stages of T cells, monocytes, myeloid cells, TBNK (T cells, B cells, natural killer cells), and Treg panels, while the MP feature contains CDC and TBNK panels. The original GWAS on immune traits was performed using data from 3,757 European individuals and there was no overlapping cohorts. Approximately 22 million SNPs genotyped with high-density arrays were imputed with Sardinian sequence-based reference panel [24] and associations were tested after adjusting for covariates (i.e., sex, age and age^2^).

### Selection of instrumental variables (IVs)

In accordance with recent research [22, 25], the significance level of IVs for each immune trait was set to 1 × 10^− 5^. The clumping procedure in PLINK software (version v1.90) was used to prune these SNPs (linkage disequilibrium [LD] *r*^2^ threshold < 0.1 within 500 kb distance) [26], where LD *r*^2^ was calculated based on 1000 Genomes Projects as a reference panel. For SCZ, we adjusted the significance level to 5 × 10^− 8^. The proportion of phenotypic variation explained (PVE) and F statistic were calculated for each IV to evaluate IV strength and avoid weak instrumental bias. A total of 7 to 1,786 independent IVs for immunophenotype were determined and these generated IVs could explain an average of 0.240% (range 0.004 − 3.652%) of the variance in their respective immune traits. Then, 108 IVs for SCZ were preserved for further analysis, after removing IVs with low F statistics (< 10).

### Statistical analysis

All analyses were performed in R 3.5.3 software (http://www.Rproject.org).

To evaluate the causal association between 731 immunophenotypes and SCZ, inverse variance weighting (IVW) [27], weighted median-based [28] and mode-based methods [29] were mainly performed by using the ‘Mendelian-Randomization’ package (version 0.4.3) [30]. Cochran’s Q statistic and corresponding *p* values were used to test the heterogeneity among selected IVs. If the null hypothesis is rejected, random effects IVW was used instead of fixed-effects IVW [27]. To exclude the effect of horizontal pleiotropy, a common method was used (i.e., MR-Egger), which implies the presence of horizontal multiplicity if its intercept term is significant [31]. Furthermore, a powerful method, the MR pleiotropy residual sum and outlier (MR-PRESSO) method was utilized to exclude possible horizontal pleiotropic outliers that could substantially affect the estimation results in the MR-PRESSO package [32]. In addition, scatter plots and funnel plots were used. Scatter plots showed that the results were not affected by outliers. Funnel plots demonstrated the robustness of the correlation and no heterogeneity.

## Results

### Exploration of the causal effect of SCZ onset on immunophenotypes

**Fig. 1:**
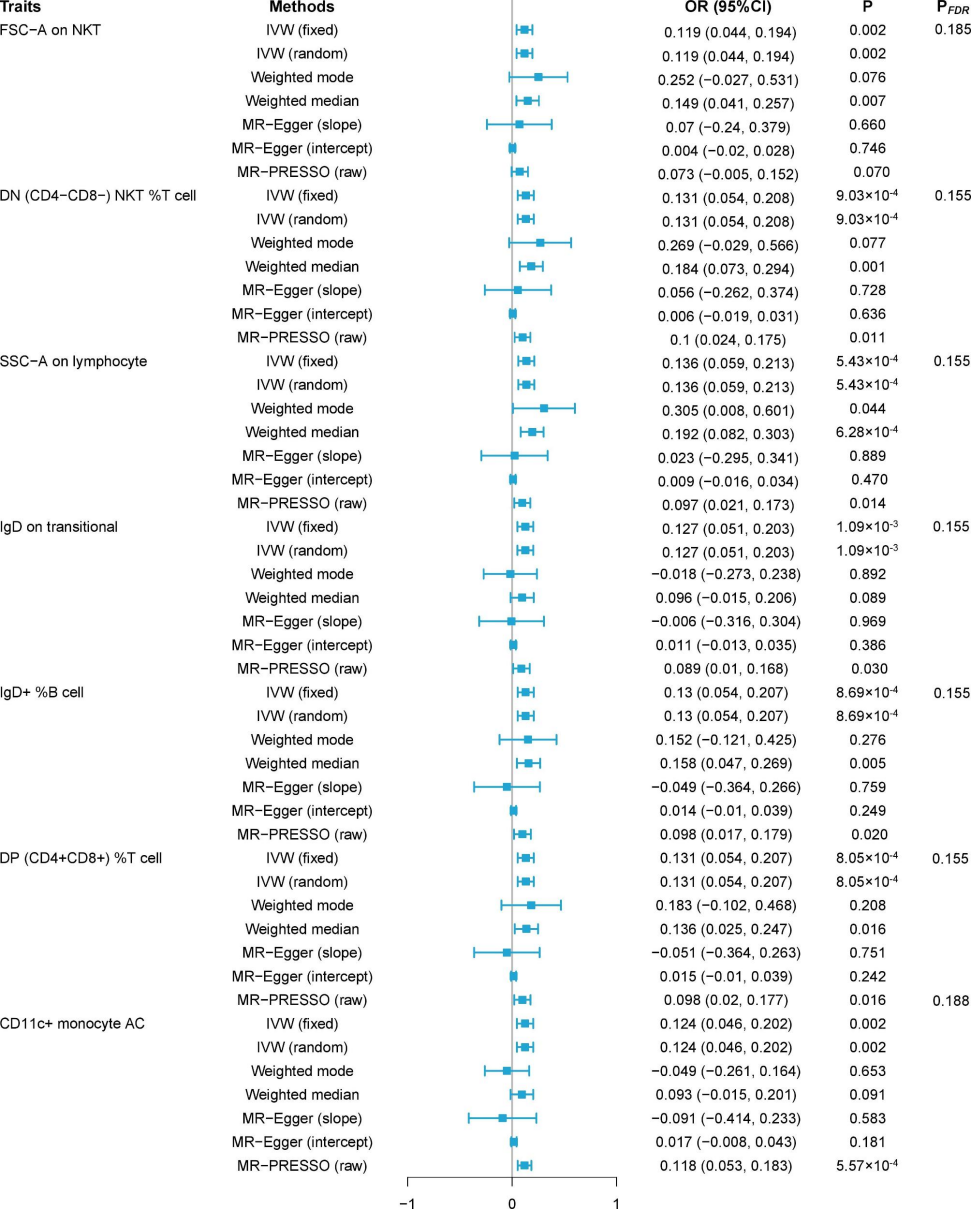
Forest plots showed the causal associations between SCZ and immune cell traits. IVW: inverse variance weighting; CI: confidence interval

To explore the causal effects of SCZ on immunophenotypes, two-sample MR analysis was performed, and the IVW method was used as the main analysis. After multiple test adjustment based on the FDR method, no immune trait was identified at a significance of 0.05. At the significance of 0.20, 7 suggestive immunophenotypes were identified, of which 2 were in the B cell panel, 1 in the cDC panel and 4 in the TBNK panel. We found that SCZ onset could increase the level of *FSC-A on NKT* (*β* = 0.119, 95% CI = 0.044 ~ 0.194, *P* = 0.002, *P*_*FDR*_ = 0.185, Fig. 1, Supplementary Tables 1, 2). *DN (CD4-CD8-) NKT %T cells* were increased in SCZ patients (*β* = 0.131, 95% CI = 0.054 ~ 0.208, *P* = 9.03 × 10^− 4^, *P*_*FDR*_ = 0.155, Fig. 1, Supplementary Tables 1, 2). *SSC-A on lymphocytes* was also found to be increased (*β* = 0.136, 95% CI = 0.059 ~ 0.213, *P* = 5.43 × 10^− 4^, *P*_*FDR*_ = 0.155, Fig. 1, Supplementary Tables 1, 2). The causal effect of SCZ on *IgD on transitional* was estimated to be 0.127 (95% CI = 0.051 ~ 0.203, *P* = 1.09 × 10^− 3^, *P*_*FDR*_ = 0.155, Fig. 1, Supplementary Tables 1, 2), but the weighted median did not support this association: weighted median (*β* = 0.096, 95% CI = -0.015 ~ 0.206, *P* = 0.089). For *IgD+ %B cell*, a positive association was observed (*β* = 0.130, 95% CI = 0.054 ~ 0.207, *P* = 8.69 × 10^− 4^, *P*_*FDR*_ = 0.155, Supplementary Table 2), which was consistent the with weighted median and MR-PRESSO, but inconsistent with weighted the mode. Similar associations were found for *DP (CD4*^*+*^*CD8*^*+*^*) %T cell* (*β* = 0.131, 95% CI = 0.054 ~ 0.207, *P* = 8.05 × 10^− 4^, *P*_*FDR*_ = 0.155, Supplementary Table 2), and *CD11c*^*+*^*monocyte AC* (*β* = 0.124, 95% CI = 0.046 ~ 0.202, *P* = 0.002, *P*_*FDR*_ = 0.188, Supplementary Table 2). The results of the other three methods and sensitivity analysis proved the robustness of the causal associations observed (Supplementary Tables 1, 3). Specifically, the intercept of MR-Egger and global test of MR-PRESSO ruled out the possibility of horizontal pleiotropy (Supplementary Table 1). Scatter plots and funnel plots also indicated the stability of the results (Supplementary Fig. 1 and Fig. 3).

### Exploration of the causal effect of immunophenotypes on SCZ

**Fig. 2:**
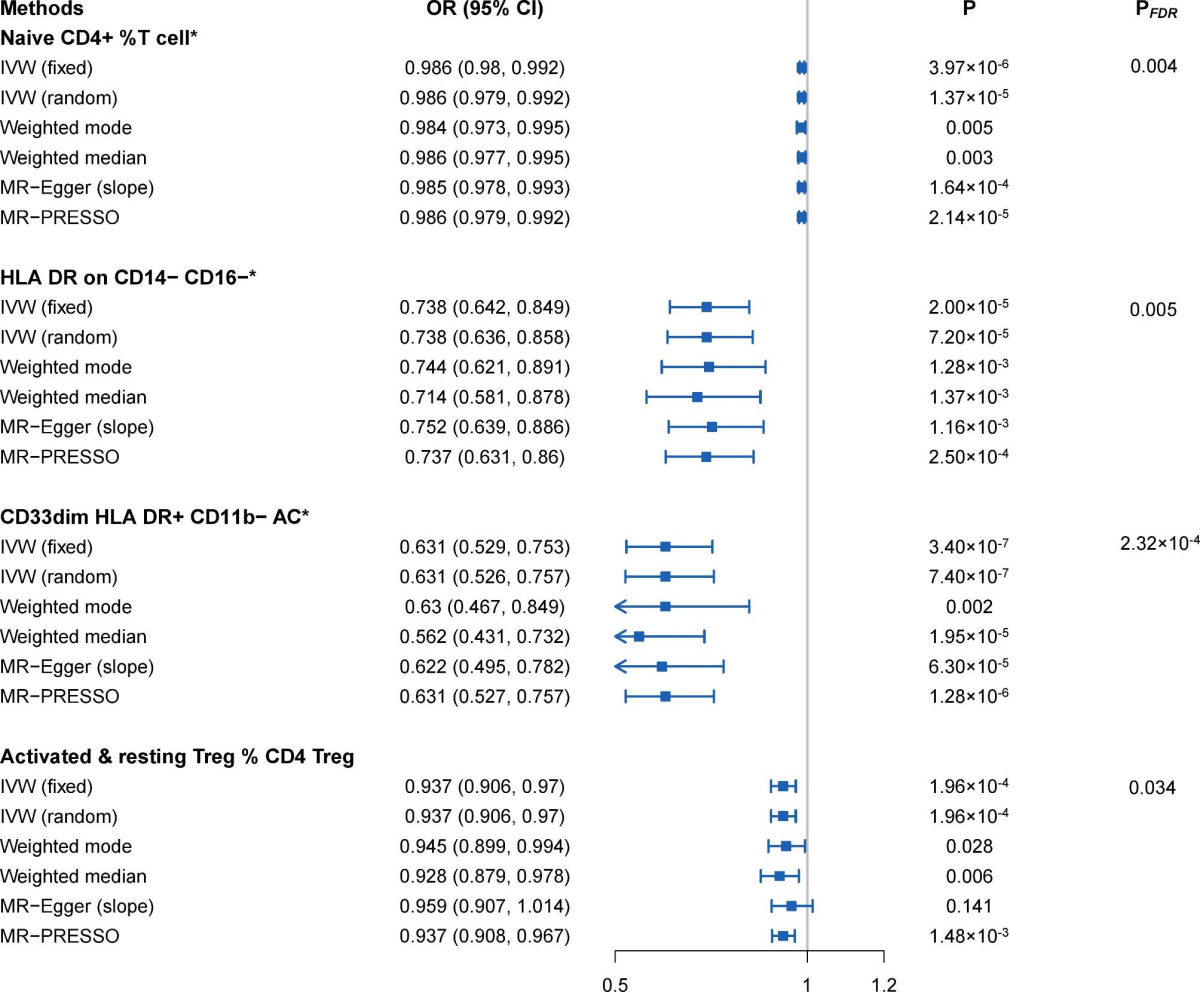
Forest plots showed the causal associations between immune cell traits and SCZ by using different methods. IVW: inverse variance weighting; CI: confidence interval

After FDR adjustment (*P*_*FDR*_<0.05), we detected protective effects of four immunophenotypes on schizophrenia: *naive CD4*^*+*^*%T cell* (maturation stages of T cell panel), *HLA DR on CD14*^*−*^*CD16*^*−*^ (monocyte panel), *CD33*^*dim*^*HLA DR*^*+*^*CD11b*^*−*^*AC* (myeloid cell panel) and *activated & resting Treg % CD4 Treg* (Treg panel). Specifically, the odds ratio (OR) of *naive CD4*^*+*^*%T cell* on SCZ risk was estimated to be 0.986 (95% CI = 0.979 ~ 0.992, *P* = 3.97 × 10^− 6^, *P*_*FDR*_ = 0.004, Supplementary Table 4) by using the IVW method. Similar results were observed by using three more methods: weighted mode (OR = 0.984, 95% CI = 0.973 ~ 0.995, *P* = 0.005); weighted median (OR = 0.986, 95% CI = 0.977 ~ 0.995, *P* = 0.003); and MR-PRESSO (OR = 0.986, 95% CI = 0.979 ~ 0.992, *P* = 2.14 × 10^− 5^). The OR of *HLA DR on CD14*^*−*^*CD16*^*−*^ on SCZ risk was estimated to be 0.738 (95% CI = 0.642 ~ 0.849, *P* = 2.00 × 10^− 5^, *P*_*FDR*_ = 0.005, Supplementary Table 4) by using the IVW method. Similar results were observed by using three more methods: weighted mode (OR = 0.744, 95% CI = 0.621 ~ 0.891, *P* = 0.001); weighted median (OR = 0.714, 95% CI = 0.581 ~ 0.878, *P* = 0.001); and MR-PRESSO (OR = 0.737, 95% CI = 0.631 ~ 0.860, *P* = 2.50 × 10^− 4^). The OR of *CD33*^*dim*^*HLA DR*^*+*^*CD11b*^*−*^*AC* on SCZ risk was estimated to be 0.631 (95% CI = 0.529 ~ 0.753, *P* = 3.40 × 10^− 7^, *P*_*FDR*_ = 2.32 × 10^− 4^, Supplementary Table 4) by using the IVW method. Similar results were observed by using three more methods: weighted mode (OR = 0.630, 95% CI = 0.467 ~ 0.849, *P* = 0.002); weighted median (OR = 0.562, 95% CI = 0.431 ~ 0.732, *P* = 1.95 × 10^− 5^); and MR-PRESSO (OR = 0.631, 95% CI = 0.527 ~ 0.757, *P* = 1.28 × 10^− 6^). The OR of *activated & resting Treg % CD4 Treg* on SCZ risk was estimated to be 0.937 (95% CI = 0.906 ~ 0.970, *P* = 1.96 × 10^− 4^, *P*_*FDR*_ = 0.034, Supplementary Table 4) by using the IVW method. Similar results were observed by using three more methods: weighted mode (OR = 0.945, 95% CI = 0.899 ~ 0.994, *P* = 0.028); weighted median (OR = 0.928, 95% CI = 0.879 ~ 0.978, *P* = 0.006); MR-PRESSO (OR = 0.937, 95% CI = 0.908 ~ 0.967, *P* = 1.48 × 10^− 3^). Additionally, both the intercept of MR-Egger and the global test of MR-PRESSO ruled out the possibility of horizontal pleiotropy for these four associations. Detailed information from the sensitivity analysis proved the robustness of the causal associations observed (Supplementary Tables 5, Fig. 2). Scatter plots and funnel plots also indicated the stability of the results (Supplementary Fig. 2).

## Discussion

Based on large publicly available genetic data, we explored causal associations between 731 immune cell traits and SCZ. To our knowledge, this is the first MR analysis to explore the causal relationship between multiple immunophenotypes and SCZ. In this study, among four types of immune traits (MFI, RC, AC and MP), SCZ was found to have causal effects on seven immunophenotypes (FDR < 0.20), and four immunophenotypes had significant causal effects on SCZ (FDR < 0.05).

Our study found that the risk of SCZ decreased with an increase in the proportion of naive CD4+%T cell. Naive CD4+% T cells are able to modulate proinflammatory and anti-inflammatory signals by differentiating into a variety of T-helper (Th) cell lineages, each with its own distinct cytokine profile and function. There is evidence that secreted cytokines play a role in the occurrence and progression of SCZ [33, 34]. Compared with healthy people, IL-6 and TNF-α levels were significantly increased in schizophrenia patients, while IL-2, IL-4 and IFN-γ levels were significantly decreased [35].

HLA DR on CD14-CD16- in the monocyte panel has been proven to be associated with decreased SCZ risk [36, 37]. HLA-DR is an MHC class II cell surface receptor encoded by the human leukocyte antigen complex on chromosome 6 region 6P21. Previous studies have shown that lower concentrations of IL-1β in cells may reflect the weakening of monocyte function in SCZ [38]. The low expression of HLA-DR in monocytes in chronic inflammation demonstrates the anti-inflammatory effect of HLA-DR molecules.

Activated and resting Treg% CD4 Tregs have also been proven to be significantly associated with a reduced risk of SCZ [39, 40]. Regulatory T cells (Tregs) are the key immunomodulatory cells involved in the control of inflammatory processes, and their function is directly related to the human leukocyte antigen (HLA) gene, which has been implicated in genetic studies of SCZ. A significantly increased proportion of Tregs in patients with SCZ compared to healthy controls. Tregs were found to be able to improve the negative symptoms of schizophrenia by offsetting the ongoing inflammatory process [41].

In addition, it was noteworthy that the presence of SCZ was found to be associated with increased FSC-A on NKT, DN (CD4-CD8-) NKT %T cell, SSC-A on lymphocytes, IgD on transitional cells, IgD+ %B cell, DP (CD4 + CD8+) %T cell, and CD11c + monocyte AC levels. Several studies have found increased concentrations of inflammatory cytokines in the blood of patients with SCZ [42]. To date, there has been limited research on the role of NKT cells in neurological diseases, and a case-control study found an increase in the relative number of NK cells in schizophrenia [43]. Notably, Finkelstein et al. reported that the function of NKT cells in neurodegeneration is limited [44]. Mechanistically, the regulation of NKT cells induces a cytokine shift in the liver and promotes the recruitment of T cells into the affected spinal cord [45].

This study conducted two-sample MR analysis based on the published results of large GWAS cohorts, with a large sample size of approximately 150,000 people, so it has high statistical efficiency. The conclusions of this study are based on genetic instrumental variables, and causal inference is made using a variety of MR analysis methods. The results are robust and were not confounded by horizontal pleiotropy and other factors. Our study has limitations. First, even when multiple sensitivity analyses are performed, horizontal pleiotropy cannot be fully assessed. Second, due to the lack of individual information, we cannot conduct further stratified analysis of the population. Third, the study was based on a European database, so the conclusion cannot be extended to other ethnic groups, which would limit the generalizability of our results. Finally, we used a looser threshold to evaluate the results, which may increase some false-positives while simultaneously enabling a more comprehensive assessment of the strong association between the immune profile and SCZ.

## Conclusions

In conclusion, we have demonstrated the causal associations between several immunophenotypes and SCZ through a comprehensive bidirectional MR analysis, highlighting the complex pattern of interactions between the immune system and SCZ.

Furthermore, our research significantly reduced the impact of unavoidable confounding factors, reverse causality, and other factors. It may provide a new avenue for researchers to explore the biological mechanisms of SCZ and can lead to exploration of earlier intervention and treatment. Our results extend the findings of psychoimmunology, providing valuable clues for the prevention of SCZ.

### Electronic supplementary material

Below is the link to the electronic supplementary material.

Supplementary file 1: Causal effects of schizophrenia on immune cells

Supplementary file 2: IVW results of the causal effect of SCZ on immune cells

Supplementary file 3: Results of the causal effect of immune cells on SCZ

Supplementary file 4: Sensitivity analysis results of causal effects of SCZ on immune cells

Supplementary file 5: Sensitivity analysis results of causal effects of immune cells on SCZ

Supplementary file 6: Supplementary figures

Supplementary file 7: Supplementary Methods

## Data Availability

The raw data supporting the conclusions of this article will be made available by the authors, without undue reservation. Further inquiries can be directed to the corresponding author.

## List of Abbreviations

AC: Absolute cell
CI: Confidence interval
FDR: False discovery rate
GBD: Global Burden of Disease
GWAS: Genome-wide association study
HLA: Human leukocyte antigen
IL-1α: Interleukin-1α
IL-1β: Interleukin-1β
IL-2: Interleukin-2
IL-6: Interleukin-6
IVs: Instrumental variables
IVW: Inverse variance weighting
LD: Linkage disequilibrium
MFI: Median fluorescence intensities
MP: Morphological parameters
MR: Mendelian Randomization
MR-PRESSO: MR pleiotropy residual sum and outlier
OR: Odds ratio
PGC: Psychiatric Genomics Consortium
PVE: Phenotypic variation explained
RC: Relative cell
SCZ: Schizophrenia
SNPs: Single Nucleotide Polymorphisms
TNF-α: Tumor necrosis factor-α

## References

[1] Khandaker GM (2014). Association of serum interleukin 6 and C-reactive protein in childhood with depression and psychosis in young adult life: a population-based longitudinal study. JAMA Psychiatry.

[2] Jauhar S, Johnstone M, McKenna PJ (2022). Schizophrenia Lancet.

[3] Charlson FJ (2018). Global epidemiology and burden of Schizophrenia: findings from the global burden of Disease Study 2016. Schizophr Bull.

[4] Correll CU. Current treatment options and emerging agents for Schizophrenia. J Clin Psychiatry, 2020. 81(3). 10.4088/JCP.MS19053BR3C 32297721

[5] Mueser KT, McGurk SR (2004). Schizophrenia Lancet.

[6] Lieberman JA, Small SA, Girgis RR (2019). Early detection and preventive intervention in Schizophrenia: from Fantasy to reality. Am J Psychiatry.

[7] Khandaker GM (2013). Prenatal maternal infection, neurodevelopment and adult schizophrenia: a systematic review of population-based studies. Psychol Med.

[8] Khandaker GM (2012). Childhood infection and adult schizophrenia: a meta-analysis of population-based studies. Schizophr Res.

[9] Benros ME (2013). Autoimmune diseases and severe infections as risk factors for mood disorders: a nationwide study. JAMA Psychiatry.

[10] Benros ME (2011). Autoimmune diseases and severe infections as risk factors for schizophrenia: a 30-year population-based register study. Am J Psychiatry.

[11] Khandaker GM (2015). Inflammation and immunity in schizophrenia: implications for pathophysiology and treatment. Lancet Psychiatry.

[12] Dantzer R (2008). From inflammation to sickness and depression: when the immune system subjugates the brain. Nat Rev Neurosci.

[13] McFarland HF, Martin R (2007). Multiple sclerosis: a complicated picture of autoimmunity. Nat Immunol.

[14] Potvin S (2008). Inflammatory cytokine alterations in schizophrenia: a systematic quantitative review. Biol Psychiatry.

[15] Fang X (2020). Association between SIRT1, Cytokines, and metabolic syndrome in Schizophrenia patients with olanzapine or Clozapine Monotherapy. Front Psychiatry.

[16] Kalayasiri R (2019). Paranoid schizophrenia and methamphetamine-induced paranoia are both characterized by a similar LINE-1 partial methylation profile, which is more pronounced in paranoid schizophrenia. Schizophr Res.

[17] Williams JA (2022). Inflammation and brain structure in Schizophrenia and other Neuropsychiatric Disorders: a mendelian randomization study. JAMA Psychiatry.

[18] Hartwig FP (2017). Inflammatory biomarkers and risk of Schizophrenia: a 2-Sample mendelian randomization study. JAMA Psychiatry.

[19] Davey Smith G, Hemani G (2014). Mendelian randomization: genetic anchors for causal inference in epidemiological studies. Hum Mol Genet.

[20] Timpson NJ, Wade KH, Smith GD (2012). Mendelian randomization: application to cardiovascular disease. Curr Hypertens Rep.

[21] Schizophrenia Working Group of the Psychiatric Genomics (2014). Biological insights from 108 schizophrenia-associated genetic loci. Nature.

[22] Orrù V (2020). Complex genetic signatures in immune cells underlie autoimmunity and inform therapy. Nat Genet.

[23] Consortium SW (2014). G.o.t.P.G., Biological insights from 108 schizophrenia-associated genetic loci. Nature.

[24] Sidore C (2015). Genome sequencing elucidates sardinian genetic architecture and augments association analyses for lipid and blood inflammatory markers. Nat Genet.

[25] Yu XH (2021). The causal role of gut microbiota in development of osteoarthritis. Osteoarthritis Cartilage.

[26] Genomes Project C (2015). A global reference for human genetic variation. Nature.

[27] Burgess S, Small DS, Thompson SG (2017). A review of instrumental variable estimators for mendelian randomization. Stat Methods Med Res.

[28] Bowden J (2016). Consistent estimation in mendelian randomization with some Invalid Instruments using a weighted median estimator. Genet Epidemiol.

[29] Hartwig FP, Davey Smith G, Bowden J (2017). Robust inference in summary data mendelian randomization via the zero modal pleiotropy assumption. Int J Epidemiol.

[30] Yavorska OO, Burgess S (2017). MendelianRandomization: an R package for performing mendelian randomization analyses using summarized data. Int J Epidemiol.

[31] Burgess S, Thompson SG (2017). Interpreting findings from mendelian randomization using the MR-Egger method. Eur J Epidemiol.

[32] Verbanck M (2018). Detection of widespread horizontal pleiotropy in causal relationships inferred from mendelian randomization between complex traits and diseases. Nat Genet.

[33] Monji A, Kato T, Kanba S (2009). Cytokines and schizophrenia: Microglia hypothesis of schizophrenia. J Neuropsychiatry Clin Neurosci.

[34] Drzyzga Ł et al. Cytokines in schizophrenia and the effects of antipsychotic drugs. Brain, behavior, and immunity, 2006. 20(6): p. 532–45. 10.1016/j.bbi.2006.02.002 16580814

[35] Na KS, Kim YK (2007). Monocytic, Th1 and th2 cytokine alterations in the pathophysiology of schizophrenia. Neuropsychobiology.

[36] Steiner J (2006). Distribution of HLA-DR-positive microglia in schizophrenia reflects impaired cerebral lateralization. Acta Neuropathol.

[37] Radewicz K (2000). Increase in HLA-DR immunoreactive microglia in frontal and temporal cortex of chronic schizophrenics. J Neuropathology Experimental Neurol.

[38] Söderlund J (2009). Activation of brain interleukin-1β in schizophrenia. Mol Psychiatry.

[39] Zamani MG (1994). Study of the possible association of HLA class II, CD4, and CD3 polymorphisms with schizophrenia. Am J Med Genet.

[40] Müller N (2000). The immune system and schizophrenia: an integrative view. Ann N Y Acad Sci.

[41] Kelly DL (2018). Increased circulating regulatory T cells in medicated people with schizophrenia. Psychiatry Res.

[42] Miller BJ (2011). Meta-analysis of cytokine alterations in schizophrenia: clinical status and antipsychotic effects. Biol Psychiatry.

[43] Fernandez-Egea E (2016). Peripheral Immune cell populations Associated with cognitive deficits and negative symptoms of treatment-resistant Schizophrenia. PLoS ONE.

[44] Finkelstein A (2011). Abnormal changes in NKT cells, the IGF-1 axis, and liver pathology in an animal model of ALS. PLoS ONE.

[45] Zhu S, Zhang H, Bai L (2018). NKT cells in liver diseases. Front Med.

