# Supplementary material for: Causal role of immune cells in schizophrenia: Mendelian randomization (MR) study
Source: BMC Psychiatry. 2023 Aug 15;23:590. doi: 10.1186/s12888-023-05081-4 (PMC10428653; doi:10.1186/s12888-023-05081-4)
Supplement: Supplementary file 6 — Supplementary figures [file 12888_2023_5081_MOESM6_ESM.pdf]

Supplementary Figures

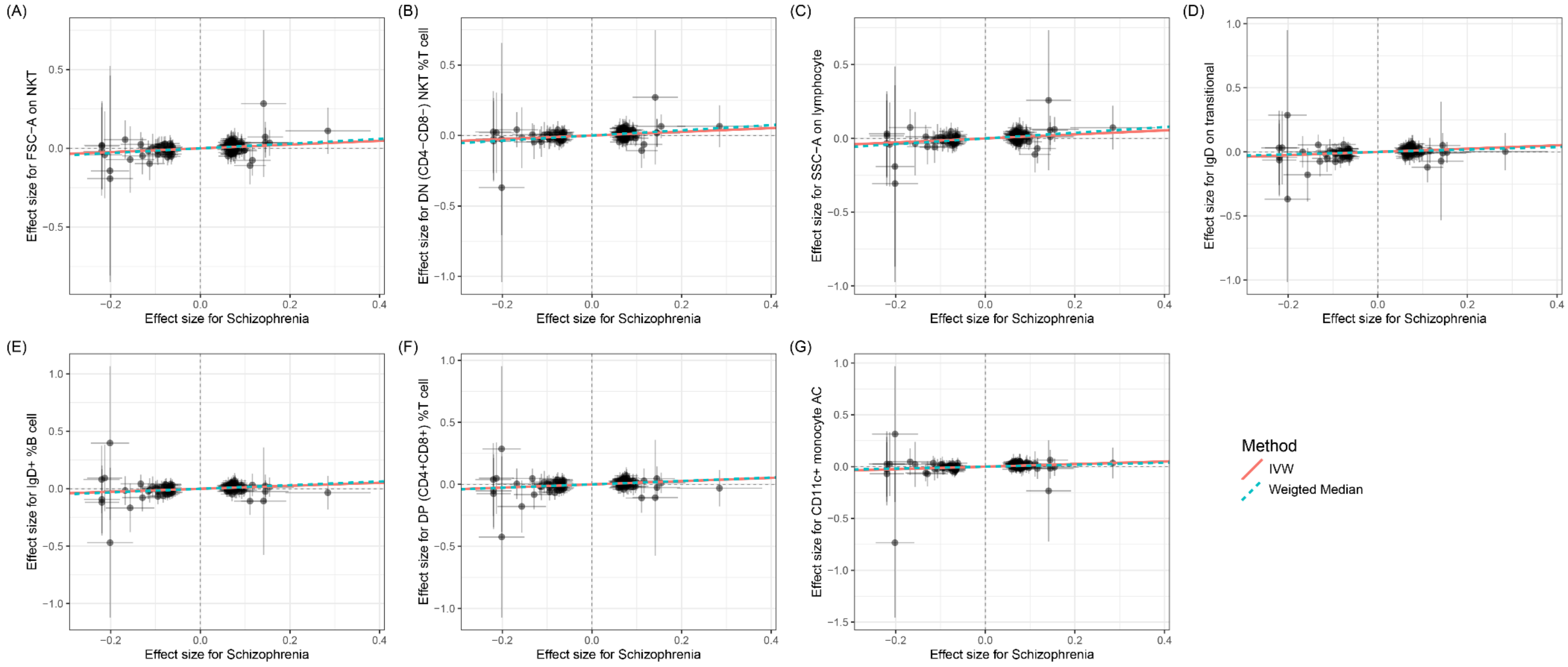

Supplementary Figure 1. Causal effects of SCZ on immune cells concentration. (A) Scatter plot between FSC-A on NKT and SCZ risk; (B)

Scatter plot between DN (CD4- CD8-) NKT and SCZ risk; (C) Scatter plot between SSC-A on lymphocyte and SCZ risk; (D) Scatter plot between IgD on transitional and SCZ risk; (E) Scatter plot between IgD+ %B cell and SCZ risk; (F) Scatter plot between DP (CD4+ CD8+)% T cell and SCZ risk; (G) Scatter plot between CD11c+ monocyte AC and SCZ risk.

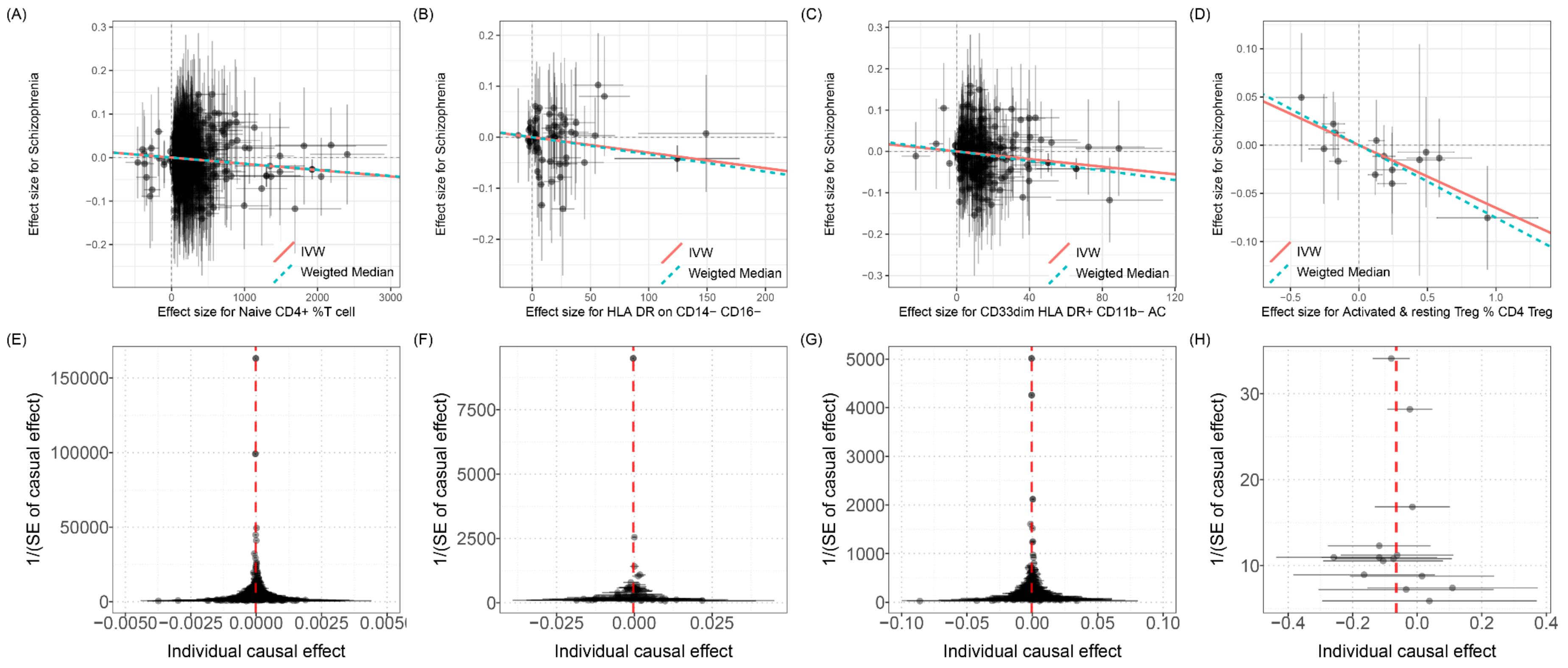

Supplementary Figure 2. Causal associations between immune cells and SCZ. (A) Scatter plot between Naïve CD4^{+} % T cell and SCZ risk; (B) Scatter plot between HLA DR on CD14- CD16- and SCZ risk; (C) Scatter plot between CD33dim HLA DR+ CD11b- AC and SCZ risk; (D) Scatter plot between Activated & resting Treg % CD4 Treg and SCZ risk; (E) Funnel plot between Naïve CD4^{+} % T cell and SCZ risk; (F) Funnel plot

between HLA DR on CD14- CD16- and SCZ risk; (G) Funnel plot between CD33dim HLA DR+ CD11b- AC and SCZ risk; (H) Funnel plot between Activated & resting Treg % CD4 Treg and SCZ risk.

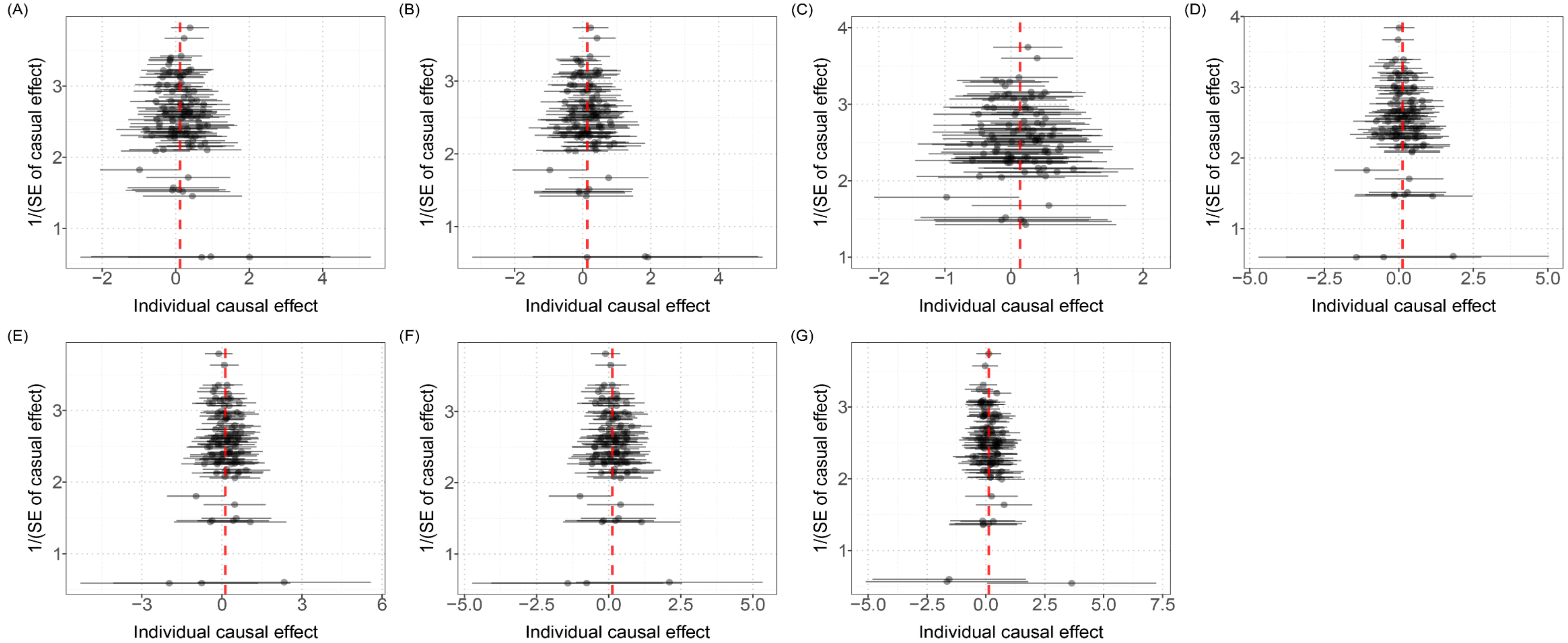

Supplementary Figure 3. Funnel plots between SCZ and immune cells.
