# Supplementary material for: Causal role of immune cells in schizophrenia: Mendelian randomization (MR) study
Source: BMC Psychiatry. 2023 Aug 15;23:590. doi: 10.1186/s12888-023-05081-4 (PMC10428653; doi:10.1186/s12888-023-05081-4)
Supplement: Supplementary file 7 — Supplementary Methods [file 12888_2023_5081_MOESM7_ESM.pdf]

Supplementary Methods

Sample exclusions and imputation were performed using the following steps:

For the genetic analyses of immune cells, a genetic map based on 6,602 samples was used, genotyped using four Illumina arrays (OmniExpress, ImmunoChip, Cardio-MetaboChip, and ExomeChip). Pre-phased genotypes were imputed genome-wide using a reference panel of 3,514 individuals and the Minimac58 software. After imputation, only SNPs with an RSQR > 0.3 (estimated minor allele frequency (MAF) ≥ 1%) or > 0.6 (MAF < 1%) were retained for association analyses, resulting in approximately 22 million variants.

For SCZ, two meta-analyses were conducted, including a first-stage analysis of cohorts with case-control phenotypes (the IGAP, ADSP, and PGC-ALZ datasets) and a proxy phenotype in the UKB cohort included in the third stage.
